# Multi-System Factors Associated with Metatarsophalangeal Joint Deformity in Individuals with Type 2 Diabetes

**DOI:** 10.3390/jcm9041012

**Published:** 2020-04-03

**Authors:** Jennifer A. Zellers, Michael J. Mueller, Paul K. Commean, Ling Chen, Hyo-Jung Jeong, Mary K. Hastings

**Affiliations:** 1 Program in Physical Therapy, Washington University School of Medicine in St. Louis, 4444 Forest Park Ave., St. Louis, MO 63108, USA; jzellers@wustl.edu (J.A.Z.); muellerm@wustl.edu (M.J.M.); jeong.h@wustl.edu (H.-J.J.); 2 Mallinckrodt Institute of Radiology, Washington University School of Medicine in St. Louis, 510 South Kingshighway Blvd., St. Louis, MO 63110, USA; commeanp@wustl.edu; 3 Division of Biostatistics, Washington University School of Medicine in St. Louis, 660 S. Euclid Ave., St. Louis, MO 63110, USA; lingchen@wustl.edu

**Keywords:** imaging, computed tomography, magnetic resonance imaging, perfusion, bone mineral density, intramuscular fat, advanced glycation end-products, hammer toe, claw toe, C-reactive protein

## Abstract

The underlying factors contributing to metatarsophalangeal joint deformity, a known precursor to skin breakdown in individuals with diabetes mellitus (DM), is likely to involve multiple body systems. The purpose of this cross-sectional study was to identify multi-system factors associated with metatarsophalangeal joint deformity in individuals with type 2 DM and peripheral neuropathy (*n* = 60). Metatarsophalangeal joint deformity was quantified with a computed tomography (CT) scan. System biomarkers included the musculoskeletal system (foot intrinsic muscle deterioration, tarsal/metatarsal bone mineral density, ankle dorsiflexion, metatarsophalangeal extension movement during a sit to stand task); the vascular system (ankle-brachial index); and the endocrine/immune systems (high sensitivity C-reactive protein, skin intrinsic fluorescence, and hemoglobin A1C). Muscle deterioration (*r* = 0.27), bone density (*r* = −0.35), metatarsophalangeal extension movement (*r* = 0.50), maximum dorsiflexion (*r* = −0.31), and ankle-brachial index (*r* = 0.33) were related to metatarsophalangeal joint deformity (*p* < 0.05). Bone mineral density and metatarsophalangeal extension movement were retained in a regression model relating to deformity (*R*^2^ = 0.34). All musculoskeletal system biomarkers and the ankle-brachial index demonstrated weak to moderate relationships to metatarsophalangeal joint deformity. Bone mineral density of the tarsal/metatarsal bones and extending the toes during a sit to stand task were the two strongest factors associated with metatarsophalangeal joint deformity. Evaluation and management of foot bone mineral density and toe extension movement pattern could reduce metatarsophalangeal joint deformity and the risk of skin breakdown and subsequent amputation.

## 1. Introduction

Diabetes mellitus (DM)-related foot complications present a substantial economic and social burden [1,2] and negatively impact individuals’ quality of life [3,4] and medical outcomes [5]. Peripheral neuropathy and metatarsophalangeal (MTP) joint hyperextension deformity are common DM-related complications that increase the risk of ulceration and amputation [6,7]. MTP joint hyperextension deformity is associated with both “claw toe” and “hammertoe” deformities, depending on the resulting flexion or extension of the interphalangeal joint. However, the area of high pressure at risk for skin breakdown is at the metatarsal head due to MTP joint hyperextension deformity, irrespective of the interphalangeal joint position. Early detection and care of foot-related complications have the potential to minimize progression and negative consequences on patients’ health and quality of life.

Diabetes is a systemic disease and MTP joint deformity is likely to be a focal manifestation of multi-system deterioration [8,9]. Neural and motor dysfunction contribute to deficits in muscle strength [10,11,12], fatty infiltration of intrinsic foot muscle [13,14,15], and altered movements during functional tasks that have been correlated to MTP joint hyperextension [16]. In addition to neural and motor dysfunction, the vascular system has a number of DM-related impairments including peripheral artery disease, endothelial dysfunction [17], and blood flow dysregulation from autonomic nervous system dysfunction [18]. Both the large and small vessels of the foot are affected, impairing muscle performance and bone mineral density [8,9,19].

Compounding neural, motor, and vascular dysfunction, chronic hyperglycemia contributes to the accumulation of advanced glycation end-products (AGEs). AGEs accumulation promotes soft tissue dysfunction and is associated with limited ankle joint mobility [14], which is thought to increase stress to the forefoot and MTP joints during weightbearing activities. Furthermore, as AGEs bind with their receptor, inflammatory pathways are upregulated, promoting a pro-inflammatory environment [20] and exacerbating cellular and tissue stress.

Impaired function of these systems is likely to occur simultaneously [8]. There is growing evidence pointing at interactions within and between systems and their role in diabetic neuropathic foot deformity. Loss of intrinsic foot muscles [13,14,15,21,22,23] has been associated with deformities in the MTP joint [13,14,22] and midfoot [15]. Foot health and amputation risk have also been found to relate to cardiovascular [24,25] and inflammatory [24] markers. For the healthcare provider, the complex interaction of body systems influencing foot health in individuals with DM and peripheral neuropathy underscores the importance of a comprehensive systems assessment [26]. However, there is limited literature characterizing the diabetic neuropathic foot across multiple body systems.

Previous research has identified independent relationships between MTP joint deformity and musculoskeletal factors (toe extension movement during sit to stand [16] and intrinsic foot muscle deterioration [13,14,22]). Study design limitations have prevented the examination of the contribution of key measures across multiple body systems hypothesized to contribute to MTP joint deformity. Therefore, the purpose of this study was to take a multi-system approach to contributors to MTP joint deformity, using a comprehensive set of clinical measures. We hypothesized that biomarkers from musculoskeletal, vascular, and endocrine/immune systems would relate to MTP joint deformity; however, we anticipated that musculoskeletal factors would bear the strongest relationship to foot deformity given that the outcome of interest is bone alignment.

## 2. Experimental Section

This is a cross-sectional analysis of individuals with type 2 DM and diabetic peripheral neuropathy from the baseline time point of an ongoing longitudinal study (ClinicalTrials.gov: NCT02616263). All data collection occurred in laboratory and clinical radiology areas at Washington University School of Medicine in St. Louis between 2016 and 2018. This study was approved by the Washington University Medical School in St. Louis, Institutional Review Board.

The presence of peripheral neuropathy was defined as an inability to sense a 5.07 Semmes-Weinstein monofilament on at least one of six locations on the plantar foot, a vibration perception threshold on the plantar aspect of the great toe greater than 25V [27], or an examination score on the Michigan Neuropathy Screening Instrument [28] greater than 2. Participants were excluded if they had peripheral neuropathy with a causal factor other than DM, severe arterial disease (ankle-brachial index (ABI) < 0.9 or > 1.3) [29,30], lower-extremity amputation involving multiple toes, acute shoulder injury (required for the aims of the longitudinal study but not relevant for this analysis), pregnancy, weight greater than 180 kg (exceeding magnetic resonance imaging (MRI) scanner weight limitations), presence of a neuropathic ulcer, or age greater than 75.

To limit bias, the individuals responsible for post-processing and measurement of data were not the same individuals as those conducting the clinical examination. 

### 2.1. Participant Characteristics

Of participants assessed for eligibility (*n* = 271), 200 did not meet inclusion criteria and 11 declined participation, resulting in 60 participants included in the study. Age, sex, body mass index, DM duration, and self-reported function per the Foot and Ankle Ability Measure (FAAM) [31] were obtained for descriptive purposes (Table 1).

### 2.2. MTP Joint Deformity Assessment

A volumetric quantitative computed tomography (CT) scan of the foot was obtained (Siemens Biograph 40 CT, Siemens Medical Systems, Inc., Iselin, NJ, USA). The participant was positioned supine with the ankle in 30˚ of plantarflexion. Scan parameters were 40 images per rotation, 370 millisecond rotation time, 24 mm collimation, 0.6 mm slice thickness, 220 mAs, 120 kVp. A CT calibration phantom (Mindways, Austin, TX, USA; Calibration value: L11G 11F1 1171 611L) was placed in front of the foot. Second MTP joint angle was measured as the angle formed by the long axis of the second metatarsal and the long axis of the second proximal phalanx. Error associated with angular measures of the foot using CT has been previously reported [32]. The supplement (180˚-angle) of the angle is reported, with a larger angle representing greater deformity [8,14].

### 2.3. Musculoskeletal System Assessment

Foot intrinsic muscle deterioration ratio was measured via MRI (Siemens Prisma Fit 3T, Siemens Medical Systems, Malvern, PA, USA) similar to those previously described [14,15,33,34]. The participant was placed supine with the extremity coil around the foot. The MRI sequence parameters were: single-shot, Dixon acquisition (Turbo Spin Echo, (TSE 2D), repetition time/echo time (TR/TE) = 1190 msec/13 msec; flip angle = 123 degrees; Field of View (FOV) = 129 × 114 mm; matrix = 512 × 576; slice thickness = 3.5 mm; pixel spacing = 0.2246 × 0.2246 mm; number of averages = 1; acquisition time was 10–13 min. Muscle and fat volumes were segmented and estimated using previously described reliable and valid measures [33]. Muscle deterioration ratio was defined as the inter/intramuscular adipose tissue volume divided by muscle volume.

Bone mineral density (BMD) of each tarsal and metatarsal bone was quantified as previously described using CT [35,36]. Each tarsal and metatarsal bone was segmented. The Hounsfield unit value for each bone was determined and converted to BMD using the calibration phantom. The mean of all 12 tarsal and metatarsal bones was used for analysis.

Ankle dorsiflexion was assessed using CT. Participants were asked to actively dorsiflex their ankle as much as possible, holding the position for the scan (up to 10 s). The ankle dorsiflexion angle was measured from the intersection of one line drawn from the bottom of the second metatarsal head to the bottom of the posterior calcaneus and a second line drawn down the middle of the tibia. 

Second MTP extension movement during a sit to stand activity was assessed using a 10 camera, 3D motion capture system (Vicon MX, Los Angeles, CA, USA) and modified-Oxford marker set [37,38]. Participants were asked to stand from a seated position in an armless chair without using their arms. Motion capture data was smoothed and MTP extension movement was measured using Visual3D (C-Motion Inc., Germantown, MD, USA) software. To measure MTP extension movement, the angle between second proximal phalanx and the second metatarsal segments was measured at peak extension and static standing. The difference between these two angles was considered the joint extension excursion. Three trials were averaged for analysis.

### 2.4. Vascular System Assessment

Ankle-brachial index (ABI) was calculated as the systolic blood pressure of the tibial artery divided by the systolic blood pressure of the brachial artery. The toe-brachial index (TBI) was determined as the first dorsal interosseous artery systolic blood pressure over the brachial systolic blood pressure.

### 2.5. Endocrine and Immune Systems Assessment

High sensitivity C-reactive Protein (hsCRP), a marker of inflammation, and hemoglobin A1C (HbA1C), a marker of glycemic control, were assessed via blood draw. Accumulation of AGEs was assessed via skin intrinsic fluorescence using a SCOUT DS (VeraLight, Albuquerque, NM, USA) skin fluorescence spectrometer [39]. Two measurements were taken from the volar aspect of the left forearm. Skin intrinsic fluorescence was excited with a light emitting diode centered at 375 nm, detected over an emission range of 375–600 nm, and is reported in arbitrary units (AU).

### 2.6. Statistical Analysis

Descriptive statistics are reported. The strength of relationship between important system measures and MTP joint deformity was examined with a Pearson correlation matrix. The variables with a significant Pearson correlation to MTP joint deformity and potential confounding demographic characteristics (age, sex, and body mass index) were considered in a multivariate linear regression analysis to quantify the association between all body system biomarkers and foot deformity. Backward model selection was used to determine which variables to retain. Regression diagnostics were used to check the assumptions of linear regression and model fit. All the statistical tests were two-sided at significance level 0.05 and analyses were performed with SAS 9.4 (SAS Inc, Cary, NC, USA). Missing data were not adjusted for in the analysis, and total *n* is reported for each variable.

## 3. Results

Descriptive statistics for body system biomarkers are listed in Table 2.

Correlations between variables are shown in Table 3. Statistically significant correlations were detected between second MTP joint angle and MTP joint extension during sit to stand, maximum ankle dorsiflexion, BMD, ABI, and muscle deterioration ratio. The relationship between second MTP joint angle and skin intrinsic fluorescence neared statistical significance (*p* = 0.05).

**Figure 1:**
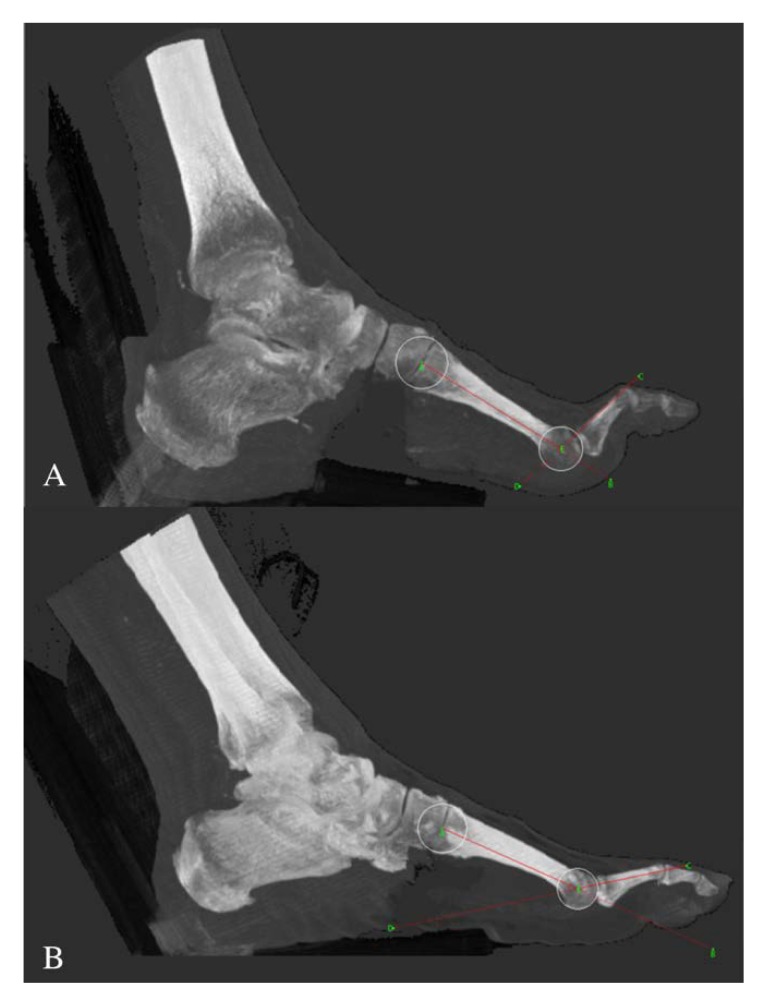
Representative CT scan images from individuals with severe (**A**, bone mineral density = 295 HU, 2nd metatarsophalangeal joint angle = 74.6 degrees) and minimal (**B**, bone mineral density = 489 HU, 2nd metatarsophalangeal joint angle = 36.1 degrees) metatarsophalangeal joint deformity. CT, computed tomography; HU, Hounsfield units.

Muscle deterioration ratio, BMD, MTP joint extension during sit to stand, maximum ankle dorsiflexion, ABI, skin intrinsic fluorescence, age, BMI, and sex were considered in a multivariate linear regression analysis to further investigate associations with second MTP joint angle. Of these variables, BMD (slope= −0.061, *p* = 0.009) and MTP joint extension movement during sit to stand (slope = 0.560, *p* < 0.001) were retained in the model. These two variables explained 34% of variation in second MTP joint angle (*R*^2^ = 0.34) (Figure 1).

## 4. Discussion

This study characterized relationships between MTP joint deformity and multi-system biomarkers in people with DM. These relationships suggest potential mechanisms underlying MTP joint deformity and future treatment targets. Lower BMD and greater amounts of MTP extension movement during the sit to stand task were the strongest factors associated with MTP joint deformity in this group of individuals. However, poor foot intrinsic muscle quality, impaired ankle dorsiflexion range of motion, and greater ABI also demonstrated weak to moderate relationships to greater MTP joint deformity. Musculoskeletal factors bear the strongest relationship to bony MTP joint deformity, but the findings of this study also support the contribution of the vascular system to foot health. Contributions from multiple body systems underscore the importance of a comprehensive clinical assessment in providing foot care to individuals with DM.

To our knowledge, this study is the first to link lower BMD with MTP joint hyperextension deformity. The importance of BMD has been established in individuals with DM and severe foot deformity, specifically Charcot neuropathic osteoarthropathy [40]. While the individuals included in this study had generally well-preserved muscle quality on MRI and good self-reported foot function per the FAAM (Table 1 and Table 2), on average this group demonstrated a 12% deficit in BMD compared to individuals without DM previously reported using the same CT technique [35]. The relationship of BMD and MTP joint deformity found in the present study is in contrast to a prior study reporting no relationship between MTP joint deformity and metatarsal head or shaft BMD [7]. It may be that isolated BMD changes close to the joint line are also influenced by arthritic or cortical bone changes that do not represent overall bone health in the foot as well as the mean BMD of all tarsal and metatarsal bones. Interventions aimed at improving BMD through modifying physical activity or medical means may be helpful in slowing the progression of MTP joint deformity, and should be considered in future, longitudinal studies.

The relationship between movement pattern and deformity underscores the importance of assessing the diabetic foot in a resting position and with movement. It has been reported that extension of the MTP joints during functional movements, such as walking and moving between sitting and standing, relates to severity of second MTP joint deformity [16]. The results of this study show a similar magnitude of relationship between MTP extension movement while moving sit to stand and severity of second MTP joint deformity. We also observed a relationship between foot intrinsic muscle quality and MTP extension movement pattern. This pattern is similar to what is observed with intrinsic wasting in the hand. As the intrinsic muscles atrophy and finger flexor muscles adaptively shorten, there is a tendency toward clawing of the fingers [41]. It seems that the foot may follow a similar pattern in which the lower leg musculature is recruited in anticipation of standing, but the foot intrinsic muscles are unable to counterbalance the pull of the toe extensors and shortening of the toe flexors. Over time, it is reasonable to speculate that abnormalities in alignment and aberrant movement patterns could advance deformity and potential for ulceration at the metatarsal heads or on the toes [13,14,16]. Future studies investigating the correction of aberrant movement patterns may help to determine whether or not interventions aimed at correcting these movement patterns can slow the progression of MTP joint deformity.

Diabetic peripheral neuropathy has been consistently associated with lower foot intrinsic muscle volume [21,22] and poor muscle quality [13,14,15]. Prior studies using quantitative measures of foot intrinsic muscle quality have identified moderate, statistically significant relationships between intrinsic muscle quality and 2nd MTJ angle [13,14]. Cheuy et al. [13] reported a possible threshold effect as the magnitude of association between muscle deterioration and second MTP joint angle increased with muscle deterioration ratios greater than 1.0. Similar to prior studies [13,14], we observed a weak, statistically significant relationship between foot intrinsic muscle quality and second MTP joint angle in the present study (*r* = 0.27). Muscle deterioration ratio (volume of intramuscular fat/muscle) was similar to healthy participants reported previously using similar methods [13,15] and lower than values previously published in individuals with DM and peripheral neuropathy [13,15], indicating well-preserved muscle quality in the individuals included in the present study. The relatively well-maintained muscle quality in the group of individuals included in the present study may have limited the magnitude of this relationship.

Vascular dysfunction has been inconsistently linked to alterations in BMD [19], though we did not see a similar relationship in this study. To our knowledge, however, this is the first study to report a relationship between macro-vasculature and MTP joint deformity. Specifically, in this group of individuals with a range of what could be considered normal ABI values [29,30], we found that individuals with higher ABI, an indicator of calcific arterial disease, had greater MTP joint deformity. It is important to note that, while the cutoff used in this study is supported by the American Diabetes Association for the diagnosis of peripheral arterial disease in people with DM [30], there is not a universally agreed upon cutoff for normal ABI [42], so individuals included in our study could have underlying calcific disease. Further, vascular dysfunction in people with DM is associated with presence of peripheral neuropathy [43]. Given that all of the participants in this study were considered to have peripheral neuropathy of varying severities, it is possible that the factors observed to be associated with changes in MTP joint deformity are moderated by peripheral neuropathy.

We did not observe any statistically significant relationships between MTP joint deformity and biomarkers assessing the endocrine and immune systems. It may be that MTP joint deformity is a process that takes a long time (months to several years) whereas the biomarkers included in this study capture function of these systems over a fairly short duration (up to several months). We did identify a moderate relationship between skin intrinsic fluorescence—a measure of AGEs accumulation in the skin – and hsCRP – a measure of inflammation. Both AGEs and C-reactive protein are known to upregulate inflammatory pathways, including the NF-kappaB pathway [20,44]. Relationships between AGEs in the blood and C-reactive protein have been reported in individuals with DM [45,46], but to our knowledge the present study is the first to report this relationship using AGEs assessed via skin intrinsic fluorescence.

This is a cross-sectional study designed to investigate relationships between factors, so change in these factors over time with disease progression is speculative. Restrictive inclusion criteria for ABI and functional limitations resulted in limited variability of these parameters within the group of individuals studied. Individuals with severe deformity, ulceration, functional limitations, and peripheral vascular disease were excluded, so the findings of this study should not be extrapolated to these individuals. The group of individuals included in this study on average had well-controlled, type 2 DM with comorbid obesity. Fifty-seven percent of the group were female. These characteristics are important to consider when extrapolating these findings to other patient groups. The CT dorsiflexion measure used is limited since it is an active range of motion measure and could be impacted by participant strength. However, the CT measure has the advantage of visualizing landmarks rather than relying on palpated landmarks, as would be the case with passive range of motion with a goniometer.

From a clinical standpoint, the results of this study provide a potential focus for assessment and interventions for the diabetic foot, though these suggestions should be further investigated in longitudinal studies. This study supports the need for a comprehensive systems assessment. In the case of MTP joint deformity, the local, musculoskeletal system had the strongest association with deformity, but vascular dysfunction may also be a factor to consider with MTP joint deformity. Focusing an examination solely on a single system could exclude potentially important treatment targets or comorbid considerations. Concerning interventions, an exercise program to increase dorsiflexion range of motion, improve performance of the foot intrinsic muscles, and minimize MTP joint excursions during functional activities may help to slow or even reverse the progression of MTP joint deformity. Despite the challenging nature of managing this chronic problem, there is growing evidence and interest in activity and exercise interventions that have a positive effect on diabetic foot related outcomes [47,48,49,50]. Additional longitudinal studies are required to identify whether or not intervening on these factors would restore or slow joint deformity.

## 5. Conclusions

Biomarkers of the musculoskeletal and vascular systems relate to second MTP joint angle, a measure of forefoot deformity. Bone mineral density and the tendency to extend the MTP joints during typical movements had the strongest association with MTP joint deformity. These results help inform evaluation and management of multi-system factors contributing to this risk factor for skin breakdown and subsequent amputation.

## Figures and Tables

**Table 1:** Participant Demographics.

Characteristic	Value ^1^
Sex (male, female)	26, 34
Age (years)	67 (6)
Body mass index (kg/m^2^)	35 (7)
Diabetes duration (years)	19 (18)
Hemoglobin A1C (%)	7.1 (1.3)
Michigan Neuropathy Score	5 (1)
Foot and Ankle Ability Measure Score	80 (20)

^1^ Values are mean (standard deviation, SD) unless otherwise noted.

**Table 2:** Descriptive statistics for primary and secondary outcomes.

Outcome	*n*	Mean (SD) ^1^
2nd MTP ^2^ joint angle (degrees)	60	53.0 (12.8)
Muscle deterioration ratio	58	0.32 (0.18)
Bone mineral density–mean of all tarsal, metatarsal bones (HU) ^3^	60	418 (58)
MTP extension movement with sit to stand (degrees)	59	16 (10)
Maximum dorsiflexion (degrees)	60	97.7 (8.1)
Ankle-brachial index	57	1.11 (0.13)
Toe-brachial index	58	1.12 (0.13)
High sensitivity C-reactive protein (mg/L)	56	3.5 (3.1)
Hemoglobin A1C (%)	59	7.1 (1.3)
Skin intrinsic fluorescence (AU) ^4^	54	2.96 (0.69)

^1^ standard deviation (SD); ^2^ Metatarsophalangeal (MTP); ^3^ Hounsfield units (HU); ^4^ Arbitrary units (AU).

**Table 3:** Pearson correlation matrix for primary variables of interest.^1^

	2nd MTP Joint Angle	MDR	BMD	MTP Extension Movement	Maximum Dorsiflexion	ABI	SIF	HbA1C
MDR ^2^	0.273 *	—	—	—	—	—	—	—
BMD ^3^	−0.352 **	−0.138	—	—	—	—	—	—
MTP extension movement ^4^	0.504 **	0.310 *	−0.179	—	—	—	—	—
Maximum dorsiflexion	−0.311 **	−0.243	0.246	−0.284 *	—	—	—	—
ABI ^5^	0.329 *	0.123	−0.067	0.138	0.068	—	—	—
SIF ^6^	−0.268	−0.062	0.014	−0.355	0.051	−0.163	—	—
HbA1C ^7^	−0.124	−0.102	0.131	−0.150	−0.033	0.059	−0.078	—
hsCRP ^8^	−0.005	−0.049	0.191	−0.097	−0.102	−0.182	0.379 **	0.129

^1^*p* < 0.05 (*), *p* < 0.01 (**); ^2^ muscle deterioration ratio (MDR); ^3^ bone mineral density (BMD); ^4^ metatarsophalangeal (MTP); ^5^ ankle-brachial index (ABI); ^6^ skin intrinsic fluorescence (SIF); ^7^ hemoglobin A1C (HbA1C); ^8^ high sensitivity C-reactive protein (hsCRP).
